# Ramifications of m6A Modification on ncRNAs in Cancer

**DOI:** 10.2174/0113892029296712240405053201

**Published:** 2024-04-09

**Authors:** Rashid Mehmood

**Affiliations:** 1 Department of Life Sciences, College of Science and General Studies, Alfaisal University, Riyadh, Kingdom of Saudi Arabia

**Keywords:** ncRNAs, m6A modification, cancer, RNA metabolism, long con-coding RNAs, microRNAs

## Abstract

N6-methyladenosine (m6A) is an RNA modification wherein the N6-position of adenosine is methylated. It is one of the most prevalent internal modifications of RNA and regulates various aspects of RNA metabolism. M6A is deposited by m6A methyltransferases, removed by m6A demethylases, and recognized by reader proteins, which modulate splicing, export, translation, and stability of the modified mRNA. Recent evidence suggests that various classes of non- coding RNAs (ncRNAs), including microRNAs (miRNAs), circular RNAs (circRNAs), and long con-coding RNAs (lncRNAs), are also targeted by this modification. Depending on the ncRNA species, m6A may affect the processing, stability, or localization of these molecules. The m6A- modified ncRNAs are implicated in a number of diseases, including cancer. In this review, the author summarizes the role of m^6^A modification in the regulation and functions of ncRNAs in tumor development. Moreover, the potential applications in cancer prognosis and therapeutics are discussed.

## 1 INTRODUCTION

N6-methyladenosine (m6A) is a common RNA modification that involves the addition of a methyl group to the adenine base of RNA molecules. It is regarded as one of the most prevalent and widely studied RNA modifications in different types of RNA molecules. Initially identified a number of years ago [1], m6A modification has received unprecedented attention recently as it is involved in a number of cellular processes, including mRNA stability, protein translation, RNA conformational changes, modulated protein-RNA interactions, and microRNA processing [2-7]. Widely described as a representative epitranscriptomic modification, m6A has been detected in a range of organisms [8-11]. In humans, it has widespread distribution and dynamics in all major adult and fetal tissues studied so far, accentuating its constitutive impacts [12, 13]. Owing to their critical roles in a number of physiological processes, it is not surprising that anomalies in m6A modification are implicated in a number of pathological conditions, including obesity, developmental defects, neuronal disorders, defective circadian clock, and cancer [14-20]. Our ability to comprehend this modification has significantly enhanced due to the development of a variety of tools that can identify it [21-31]. The last decade has observed great strides in our understanding of how this modification impacts various signaling pathways in various tissues.

Like the epigenetic modifications, m6A modification is reversible and has three groups of proteins that regulate the abundance and impacts of this modification: *Writers*, *Readers* and *Erasers* [32, 33]. The dynamic interplay among the three regulators directs the downstream functions and abundance of m6A. *Writers*, as the name implies, contribute to the deposition of the methyl group to the target RNA molecules and include a multicomponent methyltransferase complex consisting of Methyltransferase Like 3 (METTL3) [34], METTL14 [35, 36], Wilms Tumor 1 Associated Protein (WTAP) [37, 38], KIAA1429 [39], RNA Binding Motif Protein 15 (RBM15) [40], and zinc finger CCCH domain-containing protein 13 (ZC3H13) [41, 42], wherein METTL3 is the main catalytic component, while METTL14 is required for RNA substrate recognition [43-45]. WTAP is devoid of a catalytic domain and may serve as a platform for interacting with METTL3 and METTL14. *Erasers* include fat mass and obesity-associated protein (FTO) and α-ketoglutarate-dependent dioxygenase alkB homolog 5 (ALKBH5) that can actively remove the methyl group [46, 47], making this modification reversible. FTO carries out the oxidation of m^6^A to A through intermediate products in a stepwise fashion [2]. In addition to its function as a demethylase, FTO regulates alternative splicing of pre-mRNAs, alternative polyA site usage, and 3’UTR processing [48, 49]. ALKBH5 possesses m^6^A demethylation activity similar to that of FTO but operates in a sequence-specific manner for active demethylation of m6A [47]. *Readers* of m6A represent a diverse set of molecules that identify and relay the downstream signals with diverse consequences. Our understanding of the downstream effects is still evolving as we identify new molecules that bind the methylated RNA and alter its fate. Thus far, a plethora of proteins have been identified, and the list continues to increase [50]. The biological effects of m6A have been reported to be diverse depending on the molecules that bind this modification. This essentially means that the same m6A modifications may have opposite biological consequences when bound by different readers.

Among the readers, members of highly conserved YTH family proteins bind m6A-containing RNAs with different outcomes for the bound RNA molecules [51]. YTHDC1 binds m6A containing mRNAs in the nucleus and regulates exon inclusion by selectively recruiting or blocking different splicing factors to the binding regions of targeted mRNAs [52]. It also facilitates the nuclear export of m^6^A-modified mRNAs and promotes *XIST*-mediated X chromosome silencing [40, 53]. YTHDF family members are primarily cytoplasmic but have different functions, including mRNA stability, localization, and protein translation. YTHDF1, for instance, binds to m6A in the 3’ untranslated region (UTR) and recruits translation initiation machinery to promote translation [7, 54]. YTHDF2 mediates the degradation of m^6^A-containing RNA through direct recruitment of the CCR4-NOT deadenylase complex [55]. The function of YTHDF3 on m6A-modified mRNA depends on its binding partner. When bound to YTHDF1, it regulates mRNA translation [56, 57], and its direct interaction with YTHDF2 leads to m6A-modified mRNA decay [56]. However, recent evidence suggests that YTHDF proteins have redundant functions (195). YTHDC2, the structurally most complex YTH protein by virtue of its domain structure, binds a consensus motif with m^6^A and can promote translation efficiency while diminishing the mRNA abundance [58-61]. A novel function of RNA m^6^A in transcriptional regulation *via* DNA demethylation and chromatin accessibility has also been identified recently (196). Fig. (**1**) describes the roles of writers, erasers, and readers in m6A modifications and their effects on the downstream pathways.

Most of the functions of m6A modification discussed so far are related to the stability, transport, and processing of mRNA or protein translation. In addition to the above-mentioned functions of m6A on the protein-coding mRNA molecules, various non-coding RNA (ncRNAs), including microRNAs (miRNAs), long noncoding RNAs (lncRNAs), and circular RNAs (circRNAs) also serve as targets of m6A modification [62-64]. This m6A modification on lncRNAs has implications for the development of cancers, which is the focus of this review.

The following section discusses the dynamics of m6A modifications in cancer. The deregulation of the associated machinery of m6A modification, including writers, readers, and erasers in various cancers, is summarized. This will be followed by a discussion on the functions of m6A modification on ncRNAs and their effects on cancer progression.

## 2 m6A DYSREGULATION IN CANCER

m6A modification has been shown to regulate a number of physiological processes, including cell cycle regulation, ageing, and differentiation [65-67]. Various tumor suppressors and oncogenic mRNA molecules are the recipients of m6A modification [67, 68], further substantiating the connection between the modification and cancer. Any alterations in m6A in oncogenes and tumor suppressors may increase or decrease the stability of the target mRNA. For instance, m^6^A methylation on histidine triad nucleotide-binding protein 2 (*HINT2*), a tumor suppressor, is recognized by YTHDF1 that promotes its translation and significantly inhibits the progression of ocular melanoma cells in ocular melanoma [68]. Contrarily, mRNA m^6^A modification on oncogenic *CDC25B* in the M phase accelerates the translation of *CDC25B* mRNA through YTHDF1, leading to cell cycle progression and tumorigenicity in cervical cancer [67]. Many other oncogenes, including BCL-2 [69], SOX-2 [70, 71], HBXIP [19], EGFR [72], BRD4 [73], LGR5 [74], c-MYC [75], and MYB [76] are upregulated in an m6A dependent manner leading to tumor progression in multiple cancers. This dual role of targeting both the tumor suppressors and oncogenes, thus regulating both tumour progression and tumour suppression, makes the m6a modification a double-edge sword [77, 78]. Importantly, m^6^A value has been shown to predict drug response and clinical immunotherapy efficacy [79].

M6A abundance is mediated by the regulating machinery that installs and removes this modification. Additionally, the reading molecules that relay downstream signaling also contribute to the repercussions of the modification. It is not surprising that dysregulation of m^6^A-related machinery (writers, erasers, and readers) is observed in multiple cancers and has fundamental roles in cancer initiation, progression, metastasis, cancer stemness, drug resistance, and immune evasion [69, 79-81]. Upregulation of METTL3 contributes to the development and progression of prostate cancer by regulating hedgehog pathways [82] and modulating MYC methylation [83]. It also regulates the invasion and metastasis of prostate cancer cells [84]. Additionally, its upregulation is reported in other cancers with concomitant enhanced downstream oncogenic signaling [85-87]. According to multiple published articles, the components of the m6A writer complex are associated with the promotion of cancer [88-92], however, METTL14, and to some extent ZC3H13, are exceptions that act as tumor suppressors in a variety of cancers [93-96] by stabilizing PTEN mRNA and modulating EGFR/PI3K/AKT signaling pathway.

FTO, the eraser of m6A, was initially found to play an oncogenic function in acute myeloid leukemia [97]. Later studies identified its role in tumor progression in liver cancer [98], breast cancer [99], lung cancer [100], colorectal cancer [101], and cervical cancer [102]. However, tumor- suppressing functions of FTO have also been reported [103-105]. ALKBH5 has a context-dependent role and functions both as a tumor suppressor and carcinogen in different cancers and even in the same cancer type [106-111].

The readers of m6A, both nuclear and cytoplasmic, represent a diverse set of molecules with miscellaneous functions, including promoting or hampering the translation of mRNA and accelerating or inhibiting the degradation of mRNA. As these readers relay the downstream signaling, any misregulation will lead to aberrant signaling, resulting in cancer development. YTHDF1/3, for instance, has been shown to promote carcinogenesis [112, 113], while YTHDF2 has both oncogenic and tumor-suppressing functions [114, 115]. IGF2BP1-3 have also been involved with the progression of various cancers [116-118]. Other readers of m6A methylation, including YTHDC1/2, ELAVL1, and hnRNPs, also affect the progression of cancer by regulating a number of important genes implicated in tumorigenesis [119-122]. Anomalies in the expression of m^6^A regulating molecules have been associated with a poor prognosis, therapy resistance, and impaired antitumour immunity in various cancer types [123, 124].

## 3 m6A MODIFICATION ON ncRNAs IN CANCER

ncRNAs regulate a number of cellular physiological functions, including regulating target mRNA stability, splicing, processing, and transport, modulating chromatin landscape, DNA repair, and genomic stability. Research in this area continues to uncover new ncRNAs and their roles in various cellular processes, highlighting their significance in biology and medicine.

**Fig. (2):**
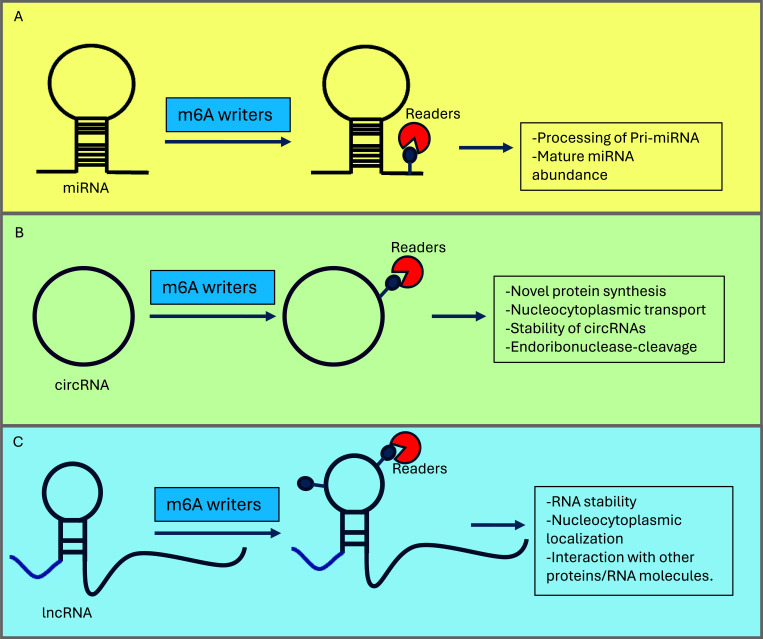
ncRNAs are also modified by m6A. (**A**) m6A on microRNAs affects their processing and abundance of mature miRNAs. (**B**) m6A on circRNAs regulates novel protein synthesis, transport, stability, and cleavage. (**C**) m6A on lncRNAs regulates their stability, localization, and interaction with other proteins/RNA molecules. M6A modification to all these ncRNAs affect the downstream signaling ultimately regulating carcinogenesis.

Like protein-coding mRNAs, ncRNAs also serve as targets for m6A modification. While a large number of ncRNAs are modified by m6A, the functions of this modification in three classes of ncRNAs, *i.e*., miRNAs, circRNAs, and lncRNAs, are discussed in the following section (Fig. **2**).

## 4 MiRNAs

MiRNAs regulate gene expression post-transcriptionally by base-pairing with target mRNAs affecting their stability or halting translation. Various miRNAs have been reported to be involved in tumorigenesis by acting as oncogenes or tumor suppressors [125]. During their biogenesis, miRNAs are initially transcribed by RNA polymerase II (Pol II) as primary-miRNA (pri-miRNA) with characteristics stem-loop structures. The pri-miRNA are initially processed in the nucleus by Drosha, an RNase III family protein, along with DGCR8 to generate pre-miRNAs which are exported out of the nucleus to the cytoplasm by Exportin-5 (EXP5). In the cytoplasm, Dicer, a member of the RNase III family protein, further processes the pre-miRNAs to generate miRNA duplex. One of the strands of the duplex is selected to carry out its downstream effects in collaboration with Argonaute (AGO) and RISC-loading complex [126]. In addition to the initial transcription by RNA Pol II, the abundance of the mature miRNAs is dependent on the processing machinery. m6A modification on unprocessed pri-miRNA by METTL3 provides an important mark that enhances DGCR8 recognition and the recruitment of processing machinery in the nucleus [5]. Depletion of METTL3 resulted in reduced binding of DGCR8 to pri-miRNAs and, depletion of mature miRNAs and concomitant buildup of unprocessed pri-miRNAs.

M6A modification on miRNAs is identified by readers that may act as inhibitors and accelerators of miRNA processing. Therefore, the abundance of oncogenic or tumor-suppressing miRNAs is linked with the activity of the machinery associated with binding m6A modification. For instance, the NF-κB activator protein NKAP interacts with DGCR8 and promotes the processing of pri-miR-25 through binding to the m6A site on pri-miR-25. M6A deposition is catalyzed by the overexpressed METTL3. Overexpressed mature miR-25 suppresses PHLPP2 which results in the activation of AKT-p70S6K signaling, which eventually leads to pancreatic cancer progression [127, 128]. HNRNPC, a direct binder of the m6A-modified site, recognizes the pri-miR-21 and promotes the expression of miR-21, which targets PDCD4, thus controlling the metastatic potential of glioblastoma [129]. HNRNPA2/B1, on the other hand, is capable of promoting and inhibiting the processing of pri-miRNAs [130]. A number of miRNAs are up or downregulated based on the reader molecules that recognize and bind to m6A modification, thus affecting the production of mature miRNA and, as a result, downstream signaling leading to inhibition or acceleration of cancer development [131-134].

The relationship between m6A and miRNA is two-ways. While m6A modification in pri-miRNA regulates their processing, the mature miRNAs also affect m6A modification by targeting mRNAs of the machinery involved in reading, writing, and erasing m6A. This adds to the complexity of molecular interactions between m6A and miRNAs. miR-33a, which acts as a tumor suppressor in several cancers [134-136], inhibits the proliferation and migration of cancer cells by targeting the 3′-UTR of *METTL3*, thus reducing the expression of METTL3 [137, 138]. In addition to METTL3, other regulators of m6A dynamics are also targeted by various miRNAs in cancer [139-142].

Adding to the complexity is the fact that miRNAs generally interact with 3’UTR of the target mRNA, a site that is also targeted by m6A methyltransferases [21]. Therefore, the target overlap might affect the ability of miRNAs to exert their effects. This target overlap is predicted to enhance post-transcriptional gene regulation by microRNAs [143, 144]. A model is proposed in which m^6^A alters the local target mRNA secondary structure to increase the accessibility of Argonaute proteins, resulting in efficient miRNA-mediated regulation [144]. This was practically demonstrated in gastric cancer, wherein a compelling role of m^6^A was identified in the post-transcriptional regulation of E2F3. m^6^A-modified motif in E2F3 was required for the interaction between E2F3 3`-UTR and miR-660 [145].

## 5 circRNAs

circRNAs form a closed loop structure due to covalent bonds between the 3' and 5' ends, leading to a circular or closed structure of these RNA molecules, unlike linear RNAs, which have a start and an end. Initially thought to be mere byproducts of splicing errors, circRNA have emerged as major players in cells where they are abundantly expressed and perform important functions [146, 147]. CircRNAs are implicated in various biological processes employing mechanisms like miRNA sponging, protein interaction, transcriptional regulation, and alternative splicing [148]. M6A modification is important as its deposition at the consensus m^6^A motifs within circRNAs can efficiently drive their translation initiation, which is driven by initiation factor eIF4G2 and m^6^A reader YTHDF3 and is augmented by methyltransferase METTL3/14 [149]. An additional function of M^6^A modification is to mediate circRNAs nucleoplasmic transport. The m^6^A readers, YTHDC1 and FMRP, are involved in the nuclear and cytoplasmic shuttling of circRNAs [150]. Deposition of m6A modification on circRNAs affect their properties and are reported in various cancers.

Several cirRNAs that are modified by m6A modulate both oncogenic and anti-oncogenic signaling pathways in various cancers. In gastric cancer, METTL14 mediates the m^6^A level and expression of circORC5 which can sponge miR-30c-2-3p to regulate AKT1S1 and EIF4B, hence promoting cancer progression [151]. M6A modified circDLC1 has been shown to inhibit MMP1-mediated liver cancer progression *via* interaction with HuR, making it a promising marker for prognosis [152]. CircMETTL3, another circular RNA enriched in m6A fraction can sponge miR-31-5p to upregulate cyclin-dependent kinases (CKD1) expression, thus promoting breast cancer progression [153]. Interestingly, the expression of circMETTL3 is regulated by its host gene, METTL3, in an m6A-dependent fashion. However, METTL3 expression is not dependent on circMETTL3 [153]. Another novel circRNA, circ1662, is highly expressed in in colorectal cancer tissues compared with the paired normal ones, and is correlated with poor prognosis. N6A-induced circ1662 promoted colorectal cancer cell invasion and migration by accelerating YAP1 nuclear transport and regulating the SMAD3 pathway [154]. m^6^A-modified circRNA, circARHGAP12, is upregulated in the cervical cancer tissue and promotes tumor progression. It promotes oncogenic signaling through m^6^A-dependent IGF2BP2/FOXM1 pathway [155]. Oncogenic role of METTL3-induced circMYO1C is also reported in PDAC tumorigenesis in an m^6^A-dependent manner where it enhances PD-L1 mRNA stability [156]. In hepatocellular carcinoma (HCC), CircMAP3K4 highly expressed. M6A modification of circMAP3K4 leads to peptide translation. The translated circMAP3K4-455aa inhibits AIF cleavage and eventually protection of HCC cells from apoptosis [157]. In contrast to these oncogenic roles, circRNAs have been reported to play the role of tumor suppressors as well.

For instance, circDLC1 overexpression inhibited glioma cell proliferation. M6A modification upregulated circDLC1 expression eventually leading to suppression of cellular proliferation [158]. circNDUFB2 inhibits non-small cell lung cancer (NSCLC) progression by degradation of IGF2BPs and activation of anti-tumor immunity [159]. These dual roles of circRNAs makes them exciting molecules for further investigation for their roles in gene regulation and therapeutic targeting [160].

## 6 LONG NON-CODING RNA (lncRNAs)

The RNA molecules longer than 200 nucleotides in length that do not code for proteins are classified as lncRNAs [161, 162]. While initially thought to be non-functional RNA, research in recent years has revealed that lncRNAs play critical roles in various cellular processes, including gene regulation, epigenetic modifications and cell cycle regulation. Dysregulation of lncRNAs has been implicated in many diseases, including cancer where they have both oncogenic and tumor-suppressive roles [163]. As important players in the complex landscape of cancer biology lncRNAs regulate cancer development and metastasis [164]; therefore, functional modification including m6A will have impact on their roles. Some lncRNAs promote oncogenic signaling by enhancing cell proliferation, blocking cell apoptosis, and facilitating cell invasion and metastasis. Examples include MALAT1, H19, HOTAIR, PVT1 and many others. Interestingly, majority of these lncRNAs undergo m6A modification that has impact on the stemness, cancer progression, metastasis and drug resistance [165-168]. Moreover, the representative lncRNAs with tumor suppressor functions including MEG3, and GAS5 are also targeted by m6A modulators [169-171]. Various lncRNAs collaborate with m6A regulating machinery to regulate a number of downstream molecules implicated in cancer development. For example, KB-1980E6.3 interacts with IGF2BP1 to facilitate m-Myc mRNA stability [172]. Contrarily, FGF13-AS1 prevents Myc mRNA stabilization by binding to IGF2BPs [173]. Similarly, lncRNA GATA3-AS guides KIAA1429 to the 3′ UTR of GATA3 pre-mRNA and facilitates in depositing m6A mark during liver cancer progression (92). Owing to their involvement in regulating important cancer related molecules, various m6A related lncRNAs have been shown to have prognostic potential [174-178].

Although lncRNAs are targeted by m6A regulating machinery and their functions are dependent on m6A methylation, several lncRNAs can also regulate writing, reading and erasing machinery involved in m6A modification. The expression of WTAP, for instance, is regulated by a number of lncRNAs including PCGEM1 in Non-small cell lung cancer [179], LINC00839 in hepatocellular carcinoma [180], SNHG10 in osteosarcoma [181], and DLGAP1-AS1in breast cancer [182]. This mutually reinforcing mechanisms wherein lncRNAs and m6A form a nexus in cancer progression also provide a target for therapeutic interventions.

## CONCLUSION

M6A modification in ncRNAs ultimately determines the structure and function of ncRNAs. We appreciate the abundance of this modification due to the development of tools that can detect it. However, despite all these studies, our understanding of modified ncRNAs in cancer is still at its infancy. Many molecular studies are needed to understand the underlying molecular mechanisms that are altered by m6A deposition on ncRNAs during cancer development. M6A modification is one of the many other modifications that RNA molecules are subjected to. How is m6A positioned in an RNA molecule in relation to other modifications needs clarifications.

The connection of m6A modification on ncRNAs and cancer development is established by numerous studies where it is shown to regulates proliferation, differentiation, metastasis, apoptosis and homeostasis. They also have critical roles in prognosis and therapy resistance. Manipulation of m6A modification by using small molecule inhibitors have just been started. A repertoire of molecules targeting writers and erasers of m6A modification have been identified with both natural and synthetic origin, and using AI approaches [183-188].

Preclinical studies highlight the potential of these small-molecule inhibitors of m^6^A modifiers with oncogenic properties in the treatment of cancer [189-191]. These molecules might have therapeutic potential either alone or in combination with conventional chemotherapy or immunotherapies, and have to be tested in cellular and animal models of cancer development. Although a number of small molecules inhibitors of writers and erasers of m6A have been developed, their non-specificity remains a formidable challenge. The non-specific effects stem from the poor specificity of the inhibitors for the target protein and the ability to modulate m6A levels globally including off target RNAs as well. Thus, the non-specificity of the inhibitors warrant caution in clinical setting. Therefore, new tools have to be established and embedded with the already existing ones. CRISPR-Cas13 is a novel powerful system that can carry out RNA editing and may be employed to regulate and edit differentially expressed and m6A modified ncRNAs to target tumors. Additionally, using larger cohorts of patients in order to identify signatures that might provide predictive and/or prognostic tools will improve our molecular understanding of cancer progression.

## Figures and Tables

**Fig. (1):**
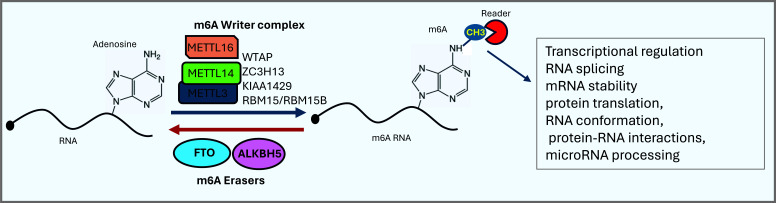
m6A modification and the regulatory proteins. Adenosine of RNA is modified by addition of methyl group “CH3” at the 6^th^ nitrogen atom of adenine by a m6A writer complex. The modification is reversible and the CH3 group is removed by m6A Erasers. The downstream effects (transcriptional and posttranscriptional) of the m6A modification vary depending on the binding proteins, called “Readers”. Various players modulating the metabolism of m6A are shown.

## LIST OF ABBREVIATIONS

AGO: Argonaute
circRNAs: Circular RNAs
EXP5: Exportin-5
HCC: Hepatocellular Carcinoma
*HINT2*: Histidine Triad Nucleotide-binding Protein 2
lncRNAs: Long con-coding RNAs
METTL3: Methyltransferase Like 3
miRNAs: microRNAs
ncRNAs: Non-coding RNAs
NSCLC: Non-small Cell Lung Cancer
RBM15: RNA Binding Motif Protein 15
UTR: Untranslated Region
WTAP: Wilms Tumor 1 Associated Protein
ZC3H13: Zinc Finger CCCH Domain-containing Protein 13
